# 5-(4-Methyl-3-nitro­phen­yl)-1*H*-tetra­zole

**DOI:** 10.1107/S1600536808018825

**Published:** 2008-07-09

**Authors:** Wei Dai, Da-Wei Fu

**Affiliations:** a Ordered Matter Science Research Center, College of Chemistry and Chemical Engineering, Southeast University, Nanjing 210096, People’s Republic of China

## Abstract

In the title compound, C_8_H_7_N_5_O_2_, the benzene ring makes a dihedral angle of 38.27 (11)° with the tetra­zole ring. The crystal structure is stabilized by N—H⋯N hydrogen bonds, forming an infinite one-dimensional chain parallel to the *a* axis.

## Related literature

For the use of tetra­zole derivatives in coordination chemisty, see: Arp *et al.* (2000 ▶); Hu *et al.* (2007 ▶); Wang *et al.* (2005 ▶); Xiong *et al.* (2002 ▶).
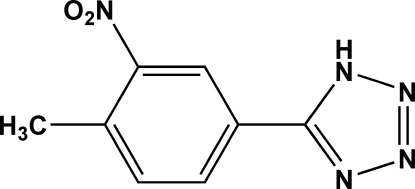


## Experimental

### 

#### Crystal data

C_8_H_7_N_5_O_2_
*M* *_r_* = 205.19
Monoclinic, 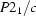

*a* = 4.9642 (10) Å
*b* = 16.982 (3) Å
*c* = 10.804 (2) Å
β = 100.71 (3)°
*V* = 894.9 (3) Å^3^
*Z* = 4
Mo *K*α radiation
μ = 0.12 mm^−1^
*T* = 298 (2) K
0.25 × 0.18 × 0.15 mm

#### Data collection

Rigaku Mercury2 diffractometer
Absorption correction: multi-scan (*CrystalClear*; Rigaku, 2005 ▶) *T*_min_ = 0.971, *T*_max_ = 0.977
7013 measured reflections
2039 independent reflections
1330 reflections with *I* > 2σ(*I*)
*R*_int_ = 0.093

#### Refinement

*R*[*F* ^2^ > 2σ(*F* ^2^)] = 0.075
*wR*(*F* ^2^) = 0.190
*S* = 1.08
2039 reflections
137 parameters
H-atom parameters constrained
Δρ_max_ = 0.32 e Å^−3^
Δρ_min_ = −0.26 e Å^−3^

### 

Data collection: *CrystalClear* (Rigaku, 2005 ▶); cell refinement: *CrystalClear*; data reduction: *CrystalClear*; program(s) used to solve structure: *SHELXS97* (Sheldrick, 2008 ▶); program(s) used to refine structure: *SHELXL97* (Sheldrick, 2008 ▶); molecular graphics: *SHELXTL* (Sheldrick, 2008 ▶) and *PLATON* (Spek, 2003 ▶); software used to prepare material for publication: *SHELXTL*.

## Supplementary Material

Crystal structure: contains datablocks I, global. DOI: 10.1107/S1600536808018825/dn2361sup1.cif

Structure factors: contains datablocks I. DOI: 10.1107/S1600536808018825/dn2361Isup2.hkl

Additional supplementary materials:  crystallographic information; 3D view; checkCIF report

## Figures and Tables

**Table 1:** Hydrogen-bond geometry (Å, °)

*D*—H⋯*A*	*D*—H	H⋯*A*	*D*⋯*A*	*D*—H⋯*A*
N1—H1*A*⋯N4^i^	0.86	2.01	2.832 (3)	160

Symmetry code: (i) 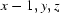.

**Fig. 1.**
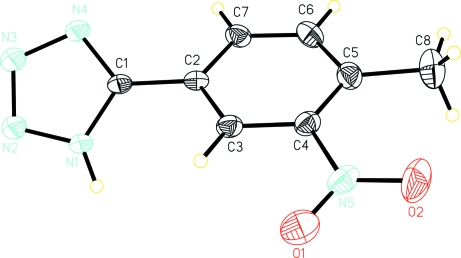
A view of the title compound with the atomic numbering scheme. Displacement ellipsoids were drawn at the 30% probability level. H atoms are represented as small spheres of arbitrary radii.

**Fig. 2.**
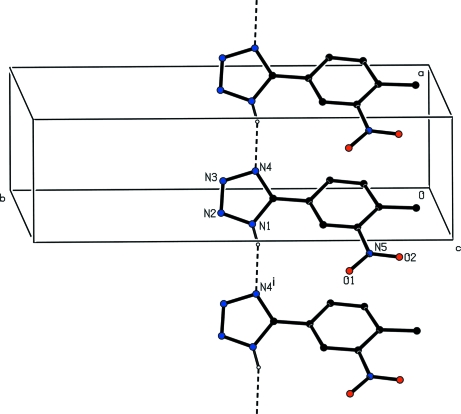
Partial packing of the title compound showing the one dimensionnal hydrogen bondings network. Hydrogen atoms not involved in hydrogen bonding (dashed lines) have been omitted for clarity. [Symmetry code : (i) x-1, y, z]

## supplementary crystallographic information

### Comment

In the past five years, our work have been focused on the chemistry of tetrazole derivatives because of their multiple coordination modes as ligands to metal ions and for the construction of novel metal-organic frameworks (Wang *et al.*, 2005; Xiong *et al.*, 2002). We report here the crystal structure of the title compound, 5-(4-methyl-3-nitrophenyl)-2*H*-tetrazole, (Fig.1).

The benzene ring makes a dihedral angle of 38.27 (0.11) ° with the tetrazole ring owing to the C–C bond bridge which force the two rings to be twisted from each other. The bond distances and bond angles of the tetrazole rings are in the usual ranges (Wang *et al.*, 2005; Arp *et al.* , 2000; Hu *et al.*, 2007). The crystal packing is stabilized by N—H···N hydrogen bonds to form an infinite one-dimensional chain parallel to the *a* axis. (Table 1, Fig. 2).

### Experimental

5-(4-methyl-3-nitrophenyl)-2*H*-tetrazole (3 mmol) was dissolved in ethanol (20 ml) and evaporated in the air affording colorless block crystals of this compound suitable for X-ray analysis were obtained.

### Refinement

All H atoms attached to C and N atoms were fixed geometrically and treated as riding with C–H = 0.9 Å (aromatic),0.96 Å(methyl) and N–H = 0.86 Å with *U*_iso_(H) =1.2Ueq(C or N).

### Crystal data

**Table:**

C_8_H_7_N_5_O_2_	*F*(000) = 424
*M**_r_* = 205.19	*D*_x_ = 1.523 Mg m^−^^3^
Monoclinic, *P*2_1_/*c*	Mo *K*α radiation, λ = 0.71073 Å
Hall symbol: -P 2ybc	Cell parameters from 2043 reflections
*a* = 4.9642 (10) Å	θ = 3.1–27.5°
*b* = 16.982 (3) Å	µ = 0.12 mm^−^^1^
*c* = 10.804 (2) Å	*T* = 298 K
β = 100.71 (3)°	Block, colorless
*V* = 894.9 (3) Å^3^	0.25 × 0.18 × 0.15 mm
*Z* = 4	

### Data collection

**Table:**

Rigaku Mercury2 diffractometer	2039 independent reflections
Radiation source: fine-focus sealed tube	1330 reflections with *I* > 2σ(*I*)
graphite	*R*_int_ = 0.093
Detector resolution: 13.6612 pixels mm^-1^	θ_max_ = 27.5°, θ_min_ = 3.1°
ω scans	*h* = −6→6
Absorption correction: multi-scan (*CrystalClear*; Rigaku, 2005)	*k* = −22→21
*T*_min_ = 0.971, *T*_max_ = 0.977	*l* = −13→14
7013 measured reflections	

### Refinement

**Table:**

Refinement on *F*^2^	Secondary atom site location: difference Fourier map
Least-squares matrix: full	Hydrogen site location: inferred from neighbouring sites
*R*[*F*^2^ > 2σ(*F*^2^)] = 0.075	H-atom parameters constrained
*wR*(*F*^2^) = 0.190	*w* = 1/[σ^2^(*F*_o_^2^) + (0.0844*P*)^2^ + 0.0553*P*] where *P* = (*F*_o_^2^ + 2*F*_c_^2^)/3
*S* = 1.08	(Δ/σ)_max_ < 0.001
2039 reflections	Δρ_max_ = 0.32 e Å^−^^3^
137 parameters	Δρ_min_ = −0.26 e Å^−^^3^
0 restraints	Extinction correction: *SHELXL97* (Sheldrick, 2008), Fc^*^=kFc[1+0.001xFc^2^λ^3^/sin(2θ)]^-1/4^
Primary atom site location: structure-invariant direct methods	Extinction coefficient: 0.037 (7)

### Special details

**Table:**

Geometry. All e.s.d.'s (except the e.s.d. in the dihedral angle between two l.s. planes) are estimated using the full covariance matrix. The cell e.s.d.'s are taken into account individually in the estimation of e.s.d.'s in distances, angles and torsion angles; correlations between e.s.d.'s in cell parameters are only used when they are defined by crystal symmetry. An approximate (isotropic) treatment of cell e.s.d.'s is used for estimating e.s.d.'s involving l.s. planes.
Refinement. Refinement of *F*^2^ against ALL reflections. The weighted *R*-factor *wR* and goodness of fit *S* are based on *F*^2^, conventional *R*-factors *R* are based on *F*, with *F* set to zero for negative *F*^2^. The threshold expression of *F*^2^ > σ(*F*^2^) is used only for calculating *R*-factors(gt) *etc*. and is not relevant to the choice of reflections for refinement. *R*-factors based on *F*^2^ are statistically about twice as large as those based on *F*, and *R*- factors based on ALL data will be even larger.

### Fractional atomic coordinates and isotropic or equivalent isotropic displacement parameters (Å^2^)

**Table:**

	*x*	*y*	*z*	*U*_iso_*/*U*_eq_	
N1	0.0809 (4)	0.47072 (13)	0.8636 (2)	0.0355 (6)	
H1A	−0.0824	0.4579	0.8719	0.043*	
N4	0.5077 (4)	0.46190 (13)	0.8535 (2)	0.0375 (6)	
C1	0.2914 (5)	0.42106 (15)	0.8715 (2)	0.0300 (6)	
C2	0.2791 (5)	0.33663 (15)	0.8959 (2)	0.0316 (6)	
N2	0.1626 (5)	0.54380 (12)	0.8406 (2)	0.0429 (6)	
N3	0.4204 (5)	0.53814 (13)	0.8340 (2)	0.0445 (6)	
C3	0.1202 (5)	0.30784 (15)	0.9784 (2)	0.0346 (6)	
H3A	0.0240	0.3423	1.0211	0.041*	
C4	0.1057 (5)	0.22713 (16)	0.9969 (2)	0.0360 (7)	
C7	0.4202 (6)	0.28333 (16)	0.8331 (3)	0.0380 (6)	
H7A	0.5288	0.3016	0.7777	0.046*	
C6	0.3991 (6)	0.20365 (16)	0.8530 (3)	0.0448 (7)	
H6A	0.4927	0.1694	0.8089	0.054*	
C5	0.2449 (6)	0.17202 (15)	0.9358 (3)	0.0423 (7)	
O1	−0.1907 (5)	0.25440 (14)	1.1326 (2)	0.0687 (8)	
O2	−0.0896 (5)	0.13387 (15)	1.1108 (2)	0.0781 (9)	
N5	−0.0701 (5)	0.20355 (16)	1.0866 (2)	0.0468 (7)	
C8	0.2416 (8)	0.08409 (18)	0.9527 (4)	0.0681 (10)	
H8A	0.3551	0.0599	0.9006	0.102*	
H8B	0.0571	0.0650	0.9289	0.102*	
H8C	0.3103	0.0713	1.0393	0.102*	

### Atomic displacement parameters (Å^2^)

**Table:**

	*U*^11^	*U*^22^	*U*^33^	*U*^12^	*U*^13^	*U*^23^
N1	0.0248 (12)	0.0353 (12)	0.0489 (14)	−0.0016 (9)	0.0130 (9)	0.0005 (9)
N4	0.0280 (12)	0.0381 (13)	0.0489 (15)	−0.0015 (9)	0.0138 (9)	0.0055 (10)
C1	0.0237 (13)	0.0375 (14)	0.0308 (13)	−0.0009 (10)	0.0101 (9)	−0.0005 (10)
C2	0.0231 (13)	0.0376 (14)	0.0350 (15)	0.0014 (10)	0.0076 (10)	−0.0001 (10)
N2	0.0352 (13)	0.0384 (14)	0.0569 (16)	−0.0006 (10)	0.0135 (11)	0.0049 (11)
N3	0.0366 (14)	0.0415 (14)	0.0587 (16)	−0.0041 (10)	0.0172 (11)	0.0038 (11)
C3	0.0295 (15)	0.0390 (15)	0.0374 (16)	0.0000 (11)	0.0120 (11)	−0.0006 (11)
C4	0.0320 (15)	0.0399 (15)	0.0363 (15)	−0.0035 (11)	0.0065 (11)	0.0057 (11)
C7	0.0349 (15)	0.0411 (15)	0.0406 (16)	0.0059 (11)	0.0141 (12)	−0.0002 (12)
C6	0.0464 (18)	0.0422 (17)	0.0466 (18)	0.0123 (13)	0.0106 (13)	−0.0044 (12)
C5	0.0426 (17)	0.0371 (16)	0.0442 (17)	0.0017 (12)	0.0005 (13)	0.0024 (12)
O1	0.0701 (17)	0.0762 (17)	0.0721 (17)	−0.0033 (13)	0.0451 (13)	0.0098 (13)
O2	0.096 (2)	0.0572 (16)	0.087 (2)	−0.0154 (12)	0.0304 (16)	0.0263 (13)
N5	0.0377 (15)	0.0574 (17)	0.0444 (15)	−0.0108 (11)	0.0053 (11)	0.0132 (12)
C8	0.091 (3)	0.0377 (18)	0.074 (3)	−0.0006 (17)	0.011 (2)	0.0031 (16)

### Geometric parameters (Å, °)

**Table:**

N1—C1	1.333 (3)	C4—N5	1.475 (3)
N1—N2	1.343 (3)	C7—C6	1.377 (4)
N1—H1A	0.8600	C7—H7A	0.9300
N4—C1	1.323 (3)	C6—C5	1.388 (4)
N4—N3	1.369 (3)	C6—H6A	0.9300
C1—C2	1.461 (3)	C5—C8	1.505 (4)
C2—C3	1.385 (3)	O1—N5	1.209 (3)
C2—C7	1.395 (3)	O2—N5	1.220 (3)
N2—N3	1.299 (3)	C8—H8A	0.9600
C3—C4	1.389 (4)	C8—H8B	0.9600
C3—H3A	0.9300	C8—H8C	0.9600
C4—C5	1.400 (4)		
			
C1—N1—N2	109.7 (2)	C6—C7—C2	120.1 (2)
C1—N1—H1A	125.2	C6—C7—H7A	119.9
N2—N1—H1A	125.2	C2—C7—H7A	119.9
C1—N4—N3	106.0 (2)	C7—C6—C5	123.2 (3)
N4—C1—N1	107.9 (2)	C7—C6—H6A	118.4
N4—C1—C2	127.1 (2)	C5—C6—H6A	118.4
N1—C1—C2	125.0 (2)	C6—C5—C4	115.1 (2)
C3—C2—C7	118.8 (2)	C6—C5—C8	118.8 (3)
C3—C2—C1	120.7 (2)	C4—C5—C8	126.0 (3)
C7—C2—C1	120.5 (2)	O1—N5—O2	122.7 (3)
N3—N2—N1	106.0 (2)	O1—N5—C4	118.4 (2)
N2—N3—N4	110.4 (2)	O2—N5—C4	119.0 (3)
C2—C3—C4	119.5 (2)	C5—C8—H8A	109.5
C2—C3—H3A	120.3	C5—C8—H8B	109.5
C4—C3—H3A	120.3	H8A—C8—H8B	109.5
C5—C4—C3	123.3 (2)	C5—C8—H8C	109.5
C5—C4—N5	122.2 (3)	H8A—C8—H8C	109.5
C3—C4—N5	114.5 (2)	H8B—C8—H8C	109.5
			
N3—N4—C1—N1	−0.1 (3)	C2—C3—C4—N5	−179.9 (2)
N3—N4—C1—C2	179.8 (2)	C3—C2—C7—C6	0.4 (4)
N2—N1—C1—N4	−0.2 (3)	C1—C2—C7—C6	−177.5 (2)
N2—N1—C1—C2	179.9 (2)	C2—C7—C6—C5	−1.1 (4)
N4—C1—C2—C3	142.9 (3)	C7—C6—C5—C4	1.2 (4)
N1—C1—C2—C3	−37.2 (4)	C7—C6—C5—C8	−178.5 (3)
N4—C1—C2—C7	−39.2 (4)	C3—C4—C5—C6	−0.6 (4)
N1—C1—C2—C7	140.7 (3)	N5—C4—C5—C6	179.2 (2)
C1—N1—N2—N3	0.4 (3)	C3—C4—C5—C8	179.1 (3)
N1—N2—N3—N4	−0.5 (3)	N5—C4—C5—C8	−1.1 (4)
C1—N4—N3—N2	0.4 (3)	C5—C4—N5—O1	−177.7 (3)
C7—C2—C3—C4	0.1 (4)	C3—C4—N5—O1	2.2 (4)
C1—C2—C3—C4	178.1 (2)	C5—C4—N5—O2	1.9 (4)
C2—C3—C4—C5	0.0 (4)	C3—C4—N5—O2	−178.3 (2)

### Hydrogen-bond geometry (Å, °)

**Table:**

*D*—H···*A*	*D*—H	H···*A*	*D*···*A*	*D*—H···*A*
N1—H1A···N4^i^	0.86	2.01	2.832 (3)	160.

Symmetry codes: (i) *x*−1, *y*, *z*.
